# Involvement of lipid microdomains in human endothelial cells infected by *Streptococcus agalactiae* type III belonging to the hypervirulent ST-17

**DOI:** 10.1590/0074-02760190398

**Published:** 2020-03-16

**Authors:** Beatriz Jandre Ferreira, Pamella Silva Lannes-Costa, Gabriela da Silva Santos, Cláudia Mermelstein, Marcelo Einicker-Lamas, Prescilla Emy Nagao

**Affiliations:** 1 Universidade do Estado do Rio de Janeiro, Instituto de Biologia Roberto Alcântara Gomes, Laboratório de Biologia Molecular e Fisiologia de Estreptococos, Rio de Janeiro, RJ, Brasil; 2 Universidade Federal do Rio de Janeiro, Instituto de Ciências Biomédicas, Rio de Janeiro, RJ, Brasil; 3 Universidade Federal do Rio de Janeiro, Instituto de Biofísica Carlos Chagas Filho, Rio de Janeiro, RJ, Brasil

**Keywords:** S. agalactiae, lipid rafts, flotillin-1, caveolin-1, HUVEC, PI3K/Akt pathway

## Abstract

**BACKGROUND:**

*Streptococcus agalactiae* capsular type III strains are a leading cause of invasive neonatal infections. Many pathogens have developed mechanisms to escape from host defense response using the host membrane microdomain machinery. Lipid rafts play an important role in a variety of cellular functions and the benefit provided by interaction with lipid rafts can vary from one pathogen to another.

**OBJECTIVES:**

This study aims to evaluate the involvement of membrane microdomains during infection of human endothelial cell by *S. agalactiae.*

**METHODS:**

The effects of cholesterol depletion and PI3K/AKT signaling pathway activation during *S. agalactiae*-human umbilical vein endothelial cells (HUVEC) interaction were analysed by pre-treatment with methyl-β-cyclodextrin (MβCD) or LY294002 inhibitors, immunofluorescence and immunoblot analysis. The involvement of lipid rafts was analysed by colocalisation of bacteria with flotillin-1 and caveolin-1 using fluorescence confocal microscopy.

**FINDINGS:**

In this work, we demonstrated the importance of the integrity of lipid rafts microdomains and activation of PI3K/Akt pathway during invasion of *S. agalactiae* strain to HUVEC cells. Our results suggest the involvement of flotillin-1 and caveolin-1 during the invasion of *S. agalactiae* strain in HUVEC cells.

**CONCLUSIONS:**

The collection of our results suggests that lipid microdomain affects the interaction of *S. agalactiae* type III belonging to the hypervirulent ST-17 with HUVEC cells through PI3K/Akt signaling pathway.

*Streptococcus agalactiae* is a leading cause of neonatal infections, such as meningitis, sepsis and pneumonia.^1^ In particular, *S. agalactiae* capsular type III strains belonging to the hypervirulent clonal complex 17 have been significantly associated with meningitis and account for up to 44 early onset disease and 67% late onset disease cases compared with less than 10% of colonising isolates.^2^ ^,^ ^3^

Microorganisms interact with host cell lipid rafts microdomains to enter and survive inside the cell.^4^ Lipid rafts play an important role in a variety of cellular functions, including polarisation, signal transduction, endocytosis, secretion, cell-cell and cell-pathogen adhesion. Several pathogens, such as viruses, bacteria and protozoa, can use the host-cell lipid rafts to secure their entrance and maintenance inside target cells. The benefit provided by interaction with lipid rafts can vary from one pathogen to another.^5^ Lipid rafts are considered as dynamic assemblies of cholesterol and sphingolipids in the plane of the membrane, resulting in an ever-changing content of both lipids and proteins.^6^ Cholesterol is a major component of microdomains, which differ from non-raft domains of the cell membrane.^7^ The cholesterol binding agent, methyl-β-cyclodextrin (MβCD), can disrupt lipid rafts by depleting cholesterol from lipid rafts and decrease the number of these specialised microdomains on the plasma membrane.^8^ Signaling molecules, including PI3Ks, are involved in cytoskeleton reorganisation, compartmentalised in lipid rafts, and are concentrated at membrane ruffles.^9^ ^,^ ^10^

The ability of *S. agalactiae* to invade a number of host-cell types has been clearly demonstrated.^1^ ^,^ ^11^ However, the invasion process is not well understood. Subversion of the PI3K/Akt pathway by *S. agalactiae* resulted in coordination of actin rearrangement and internalisation of the microorganism.^11^ PI3K is the major activator of Akt, playing a central role in fundamental biological processes including cell growth, proliferation, migration and survival, through phosphorylation of a plethora of substrates.^12^ Previous studies showed that the integrity of lipid rafts and PI3K activity are required for *S. agalactiae* invasion to Ishikawa cells.^9^ However, further studies are needed to elucidate the involvement of lipid raft components and PI3K/Akt signalling pathway during invasion of human endothelial cells by *S. agalactiae*. This work provides further evidences that lipid rafts and PI3K are implicated in *S. agalactiae* invasion to human endothelial cells.

## MATERIALS AND METHODS

*Bacterial strain and growth conditions* - *S. agalactiae* capsular type III [GBS90356 cerebrospinal fluid (CSF) strain] belonging to the hypervirulent ST-17 lineage isolated in Brazil from a 3-day-old male baby with fatal acute meningitis was used in this study. Microorganism was identified as group B streptococci and typing by methods previously described.^13^ GBS90356 isolate was cultured on blood agar base (BAB; Oxoid, Cambridge, UK) plates containing 5% sheep defibrinated blood for 24 h at 37ºC and then grown in Brain Heart Infusion broth (BHI; Difco Laboratories Inc, Detroit, MI, USA) at 37ºC until an optical density (OD) of 0.4 at ƛ = 540 nm (~10^8^ CFU/mL) was reached.^11^

*HUVEC culture -* Primary HUVEC were obtained by treating umbilical veins with 0.1% collagenase IV solution (Sigma Chemical Co., St. Louis, MO, USA) as previously described.^11^ Cells were used during first or second passages only, and subcultures were obtained by treating the confluent cultures with 0.025 % trypsin/0.2 % EDTA solution in phosphate-buffered saline (PBS) (150 mM NaCl, 20 mM phosphate buffer, pH 7.2 ― all from Sigma Chemical Co., St. Louis, MO, USA).

*Bacterial binding and intracellular viability assays* - Confluent cultures of HUVEC cells were pre-treated or not with MβCD (2 mM, Sigma Chemical Co., St. Louis, MO, USA), a lipid raft disruptor for 1 h or with LY294002, PI3K inhibitor (5 µM, Sigma Chemical Co., St. Louis, MO, USA), or with both MβCD and LY294002 for 15 min at 37ºC. Then, HUVEC were allowed to interact with *S. agalactiae* (MOI, 1:100 HUVEC/bacteria) during different periods of incubation (1, 2 and 4 h) in 5% CO_2_ at 37ºC. For the bacterial binding assays, infected monolayers were rinsed three times with M199 and lysed in a 0.5 mL solution of 25 mM Tris, 5 mM EDTA, 150 mM NaCl and 1% Igepal (all from Sigma Chemical Co., St. Louis, MO, USA). The viability of total bacteria (intracellular plus surface adherent) was estimated by plating endothelial lysates and counting the resulting colonies emerging in BAB plates containing 5% sheep defibrinated blood. To measure bacterial internalisation, the infected monolayers were rinsed three times with M199 medium and incubated for an additional 2 h period in M199 containing bactericidal amounts of gentamicin (100 μg/mL, Sigma Chemical Co., St. Louis, MO, USA) and penicillin G (5 μg/mL, Sigma Chemical Co., St. Louis, MO, USA). We also performed a count of cells that invaded and adhered shortly after the interaction with *S. agalactiae* and 0.5 h after. The number of internalised bacteria was determined as outlined above. Adherence rates were determined as: [CFU of total cell-associated (intracellular viable plus surface adherent) *S. agalactiae* - CFU intracellular *S. agalactiae*].^11^ Untreated HUVEC were used as negative control.^9^ All experiments were repeated three times.

*Field emission scanning electron microscopy (FESEM)* - HUVEC monolayers were infected with *S. agalactiae* for 2 h, washed with PBS and incubated overnight at 4ºC in a solution of 3% paraformaldehyde plus 2.5% glutaraldehyde made in 0.1 M cacodylate buffer. The strains were washed and post-fixed in a solution of 1% O_s_O_4_ plus 8 mM potassium ferrocyanide and 10 mM CaCl_2_ in 0.1 M cacodylate buffer. After washing eight times with PBS, infected cells were dehydrated in a graded series of ethanol, and the surface of some infected monolayers were scraped with scotch tape in order to expose the inner organisation of HUVEC. All cells were dried to a critical point with CO_2_ and coated with a thin gold layer. The gold-coated strains were then observed in a JEOL field emission scanner, operating at 10 kV.^14^

*Fluorescence confocal microscopy* - HUVEC cells pretreated or not with MβCD were infected with *S. agalactiae* for 1 h, rinsed with PBS and fixed with 4% paraformaldehyde in PBS for 10 min at room temperature. The cells were permeabilised with 0.5% Triton-X 100 in PBS for 30 min and incubated with primary antibodies [anti-*S. agalactiae* or anti-flotillin-1 antibody (clone 29) or anti-caveolin-1 or anti-caveolin-2] for 1 h at 37ºC. After incubation, cells were washed for 30 min and incubated with Alexa Fluor 488 or Alexa Fluor 546-conjugated secondary antibodies for 1 h at 37ºC. Nuclei were labeled with 0.5 μg/mL 4’-6-diamidino-2-phenylindole (DAPI). Cells were mounted in ProLong Gold antifade reagent and examined using a Zeiss Axiovert 100M laser confocal microscope (Carl Zeiss, Germany) by using filters sets that were selective for each fluorochrome wavelength channel. Images were acquired with a C2400i integrated charge-coupled device camera (Hamamatsu Photonics, Shizuoka, Japan) and an Argus 20 image processor (Hamamatsu). Control experiments with no primary antibodies showed only faint background staining (data not shown). All reagents and antibodies were obtained from Molecular Probes (USA). These experiments were repeated three times.

*Immunoblot analysis* - HUVEC monolayers were infected during different times with *S. agalactiae* as described above. Following infection, the plates were chilled, and all subsequent steps were carried out at 4ºC. The HUVEC were rinsed with PBS containing 0.4 mM Na_3_VO_4_ and 1 mM NaF per mL. Next, the infected cells were scraped from the plate, ressuspended in 1.5 mL of the same buffered solution, collected by centrifugation for 1 min at 12,000 g, and lysed for 30 min in 100 µL of 50 mM Tris-HCL (pH 7.6) containing 0.4 mM Na_3_VO_4_, 1 mM NaF, 1% Triton X-100, 100 µM of phenylethylsulphonylfluoride, 40 µM of leupeptin and 2 mM EDTA. The proteins were quantified, and 30 µg of protein of each extract was subjected to electrophoresis in 12% polyacrylamide separating gel (SDS-PAGE). Proteins were transferred to nitrocellulose membranes (Biorad), which were blocked and then incubated with primary antibodies. The membranes were incubated with second antibody peroxidase-conjugated and the immunoreactivity was detected using an ECL Plus detection kit (Amersham Biosciences, Buckinghamshire, UK). Autoradiographs were quantified by scanning densitometry, and the resulting absorbance curves were integrated by using the Scion Image Master. Densitometric analyses were performed on gels with different exposure times, and the ones giving linear absorbance curves were used to obtain semi quantitative assessment.^11^ These experiments were repeated three times.

*Statistical analysis* - The values of different treatments were compared using Student’s *t*-test and analysis of variance (ANOVA), followed by *Bonferroni* t test for unpaired values. All of the statistical analyses were performed at the p < 0.05 level of significance.

## RESULTS

**Fig. 1:**
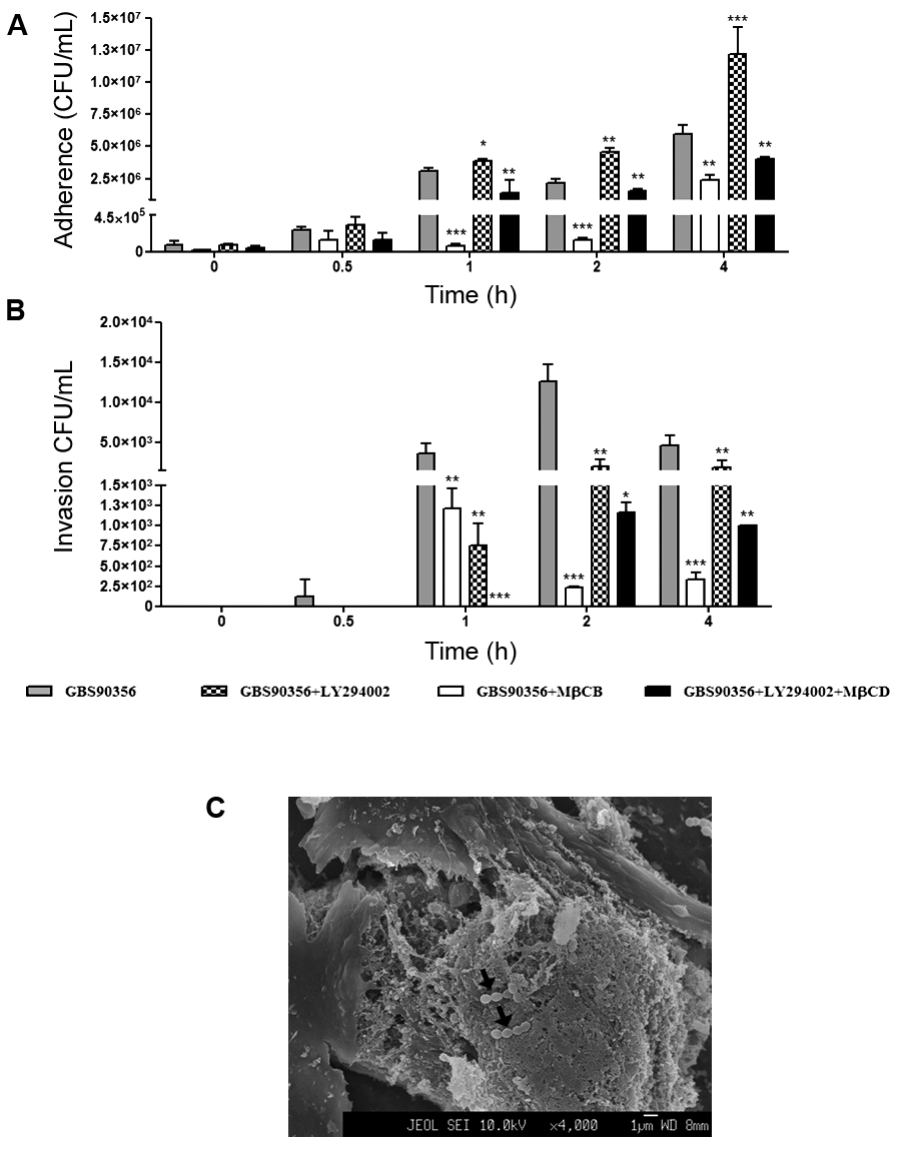
effect of methyl-β-cyclodextrin (MβCD) and PI3K inhibitors on adherence and invasion of *Streptococcus agalactiae* GBS90356 strain to human endothelial cells (HUVEC). (A) Adherence to and (B) intracellular viability of *S. agalactiae* in pre-treated HUVEC with LY294002 inhibitor of PI3K. GBS90356 strain was tested for the ability to invade human cells pretreated with MβCD or/and LY294002. (C) Arrow indicates presence of intracellular GBS90356 strain through field emission scanning electron microscopy (FESEM). Results are expressed as means ± standard deviation (SD), relative to untreated HUVEC obtained from 3 experiments. 2way analysis of variance (ANOVA), post test Bonferroni against control. Asterisk indicates *p < 0.05, **p < 0.01, ***p < 0.001.

*Effect of cholesterol depletion and PI3K inhibitor during S. agalactiae* GBS90356*-HUVEC interaction* - Effects of MβCD (cholesterol depletion agent) and LY294002 (PI3K inhibitor) treatments on *S. agalactiae* GBS90356 adherence to and invasion of HUVEC are displayed in Fig. 1. Cholesterol depletion affected bacterial binding to HUVEC 1 h post-infection (6.3 x 10^4^ CFU/mL, p < 0.001). A higher number of adherent bacteria (3.8 x 10^6^ CFU/mL, p < 0.001) was observed in HUVEC pre-treated with LY294002 in 1h and mainly after 4 h incubation (1.2 x 10^7^ CFU/mL, p < 0.001) (Fig. 1A). However, a significant reduction of *S. agalactiae* cytoadhesion was observed in HUVEC treated with LY294002 + MβCD at all chosen times of incubation (1.3 x 10^6^ CFU/mL in 1 h; 1.5 x 10^6^ CFU/mL in 2 h; 3.9 x 10^6^ CFU/mL in 4 h, p < 0.01) (Fig. 1A). *S. agalactiae* strain exhibited a strong invasive phenotype to HUVEC after 2 h post-infection. Moreover, pre-treatment of HUVEC with MβCD and/or LY294002 led to a decrease in invasion (p < 0.01) (Fig. 1B). The FESEM was used to demonstrate the presence of intracellular GBS90356 strain (Fig. 1C). Inhibition assays with MβCD and LY294002 suggest the involvement of lipid rafts and PI3K/AKT pathway during *S. agalactiae* GBS90356 internalisation process in human endothelial cells.

**Fig. 2:**
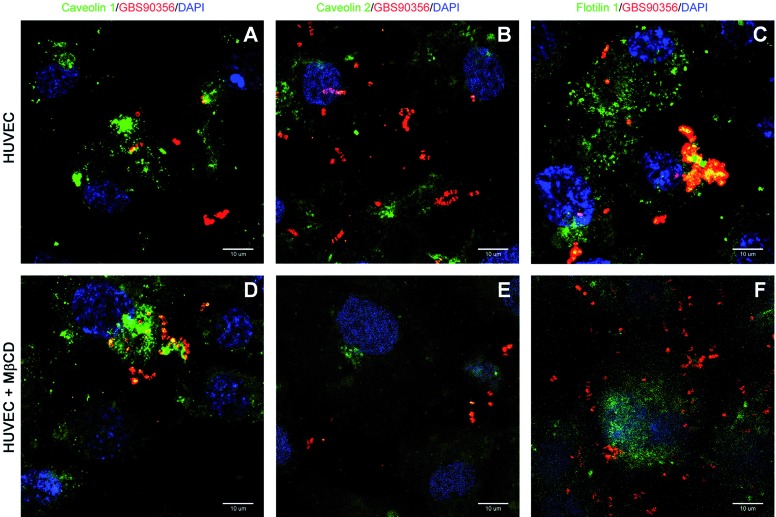
distribution of lipid rafts in human endothelial cells (HUVEC) infected with *Streptococcus agalactiae* GBS90356 strain. (A-C) HUVEC infected with GBS90356 strain (Alexa Fluor 546-conjugated; red) during 1h and labeled with anti-lipid rafts proteins (Alexa Fluor 488-conjugated; green). The nuclei were stained with DAPI (blue). D-F, HUVEC treated with 5 mM methyl-β-cyclodextrin (MβCD) and infected with GBS90356 strain and labeled with anti-lipid rafts proteins show dispersion of aggregates.

*Colocalisation of S. agalactiae GBS90356 with flotillin-1 and caveolin-1* - Cellular localisation of *S. agalactiae* GBS90356 strain, caveolin-1 (Fig. 2A, D), caveolin-2 (Fig. 2B, E) and flotillin-1 (Fig. 2C, F) in HUVEC are demonstrated by immunofluorescence microscopy. Staining for flotillin-1 revealed a colocalisation with GBS90356 strain in untreated HUVEC cells (Fig. 2C), but no colocalisation was detected in cells treated with MβCD, a cholesterol depleting agent (Fig. 2F). We also found a colocalisation of GBS90356 strain and caveolin-1 after pretreatment of HUVEC with MβCD (Fig. 2D), but not in untreated cells (Fig. 2A). By contrast, no significant colocalisation could be observed between GBS90356 strain and caveolin-2 in HUVEC cells treated or not with MβCD (Fig. 2B, E). These results suggest that caveolin-1 and flotillin-1 could be involved in the invasion of *S. agalactiae* GBS90356 strain in HUVEC cells.

**Fig. 3:**
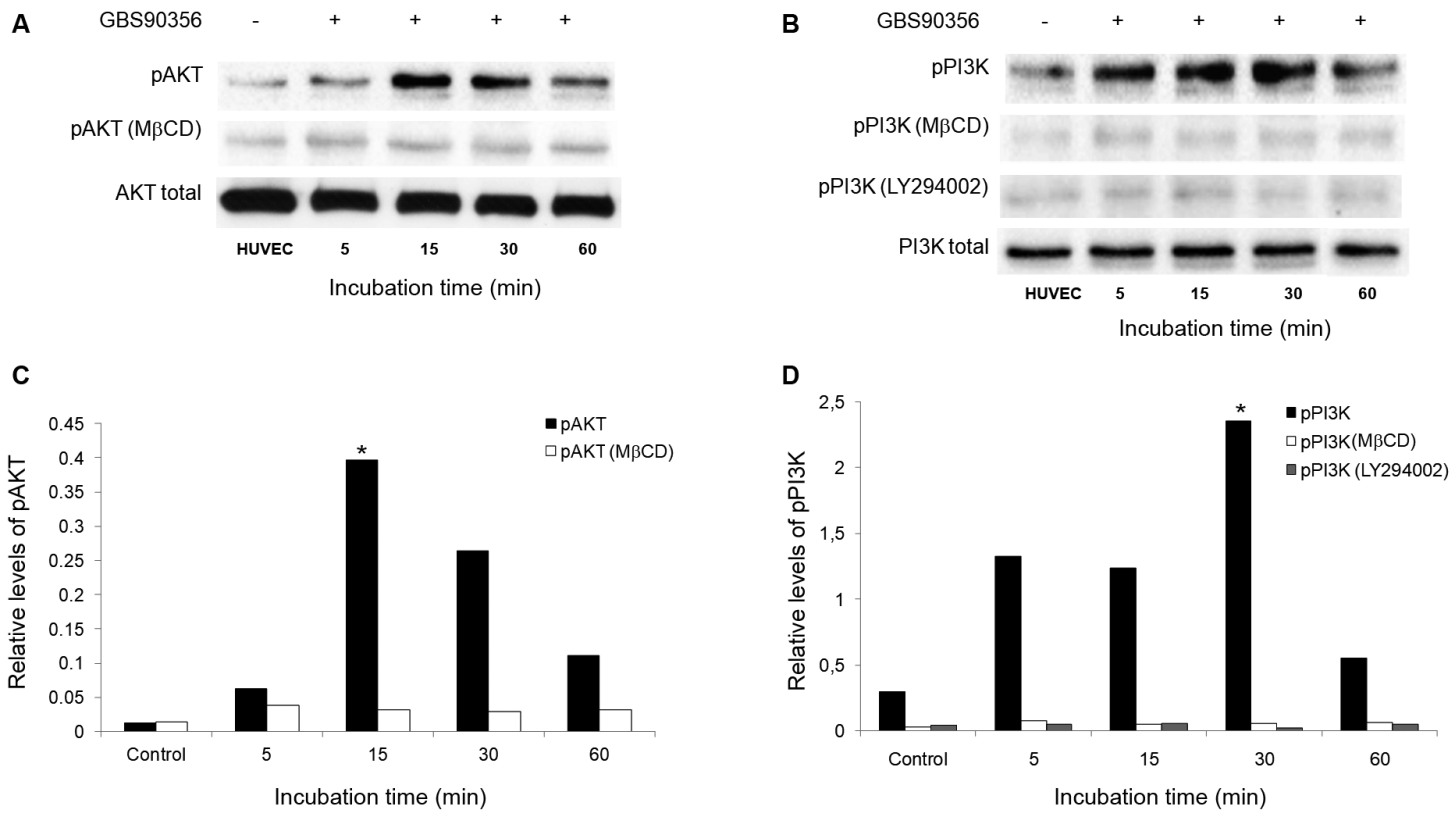
influence of PI3K/Akt pathway on *Streptococcus agalactiae*-human endothelial cells (HUVEC) interaction. (A) Immunoblots demonstrating the presence of phosphorylated Akt in HUVEC infected with GBS90356. The panel displayed the immunoreactive bands corresponding to Akt and phospho-Akt. Phospho-Akt was detected in lysates of HUVEC infected with GBS90356 for 5, 15, 30 and 60 minutes (peak 15 min). (B) Imunoblotting assay showed a significant increase in phosphorylated PI3K expression at 5 min to 30 min bacterial post-infection (peak 30 min). Both inhibitors methyl-β-cyclodextrin (MβCD) (cholesterol depletion) and LY294002 (PI3K inhibitor) abolished phospho-Akt and phospho-PI3K in infected HUVEC. The densitometric analysis of immunoreactive bands showed statistically significant differences in the amount of phospho-Akt (C) or phosphor-PI3k (D) between untreated and inhibitors treated HUVEC. *p < 0.05.

*AKT activation in HUVEC cells during S. agalactiae GBS90356 infection* - Fig. 3 shows the activation of PI3K/Akt pathway during the interaction between *S. agalactiae* GBS90356 and HUVEC cells pretreated with LY294002 (PI3K inhibitor) and/or MβCD (cholesterol depletion agent). Immunoblotting analysis revealed higher levels of phosphorylated Akt with a peak at 15 min post-infection of HUVEC by *S. agalactiae*. Inhibition assays with MβCD completely abolished the AKT phosphorylation (Fig. 3A). To verify whether the phosphorylation of Akt in HUVEC cells, treated or not with MβCD, was PI3K-dependent or -independent following infection with GBS90356 strain, the specific PI3K inhibitor LY294002 was incubated prior to bacterial infection. Activation of the PI3K pathway occurred at 5 min post-infection and peak at 30 min. Both inhibitors reduced the PI3K phosphorylation (Fig. 3B). Results were confirmed by densitometry analysis (Fig. 3C, D). Overall, the results indicate the involvement of lipid rafts and PI3K/AKT pathway activation during *S. agalactiae* internalisation in human endothelial cells.

## DISCUSSION

Lipid rafts often serve as an entry site for many microorganisms. Studies have shown that extraction of membrane cholesterol inhibited bacterial infection in the early stages of invasion.^15^ The mechanisms that underlie this interaction are starting to be unraveled. Several pathogenic bacteria have been associated with lipid rafts, such as *Francisella tularensis*, *Helicobacter pylori*, *Pseudomonas gengivalis* and *Mycobacterium tuberculosis*.^5^ Seveau et al.^16^ demonstrated for the first time that the cell adhesion molecule E-cadherin is required in host lipid rafts to mediate *Listeria monocytogenes* entry. A previous study showed that *S. agalactiae* exploited lipid rafts to invade human endometrial cells.^9^ In this work, we evaluated the influence of host cell lipid rafts during *S. agalactiae* internalisation in HUVEC by using methyl-β-cyclodextrin (MβCD), a water-soluble cyclic oligosaccharide that depletes membrane cholesterol and disrupt lipid rafts.^8^

Cholesterol-enriched membrane microdomains may provide a platform to concentrate receptors on the host cell membrane.^17^ Our data support the notion that lipid rafts on the plasma membrane of HUVEC cells facilitate entry of *S. agalactiae* GBS90356 strain. The significant decrease in cytoadhesion of GBS90356 strain to human endothelial cells treated with MβCD indicated that cholesterol depletion from the cell membrane perturbed the attachment of bacteria and altered the GBS90356 entry at post-binding steps. The reduction in the number of GBS90356 in cholesterol-depleted cells probably occurred at initial steps of infection, since significant inhibitory effect was reduced after 4h post-infection and became undetectable at 24 h post-infection (data not shown). Interaction of GBS90356 strain with cholesterol-enriched microdomains occurred probably with the participation of flotillin-1 and caveolin-1-enriched membrane microdomains.

Flotillins are present at the plasma membrane and endosomal structures and have been implicated in many cellular processes, such as lipid raft formation, cellular migration and adhesion, cell polarity, signaling by receptor tyrosine kinases and mitogen activated protein kinases (MAPK), as well as membrane trafficking.^18^ ^,^ ^19^ ^,^ ^20^ Several cargo molecules, such as the GPI-anchored protein CD59, cholera toxin B subunit, virus, proteoglycans and proteoglycan bound ligands have been suggested to utilise an internalisation pathway that depends on flotillin.^21^ ^,^ ^22^ Previous data suggested that the highly dynamic flotillin microdomains become static just prior to their internalisation, which might be caused by coalescence of flotillin oligomers into larger oligomeric structures, participating in the formation of specific non-caveolar microdomains.^23^ Currently, our results suggest that *S. agalactiae* GBS90356 induces flotillin-1 assembly to specific flotillin microdomains, which induce membrane curvature and thus generate membrane buds to entry to the HUVEC. Interestingly, the enrichment of flotillin-1 on post-LAMP endocytic organelles during maturing phagosomes might be involved in actin filament remodeling and lipid changes for phagosome-lysosomes fusion.^18^ Further studies to verify if flotillin-1 colocalise with *S. agalactiae* in early endosomes are in progress.

Interestingly, treatment with MβCD decreased the colocalisation of GBS90356 strain with flotillin-1, favoring bacterial interaction with caveolin-1. Cholesterol levels are important for maintaining membrane fluidity, and its removal can reduce lateral diffusion within the cell membrane. The reduction in fluidity could perhaps affect distribution of receptors within the plasma membrane, which may damage signal transduction events, polarisation and F-actin polymerisation.^24^ Our results suggest that cholesterol depletion by MβCD may have exposed caveolin-1 molecules, favoring recognition by GBS90356 strain. Caveolin-1 is critical for enhancing the innate immune response, which contributes to survival during LPS-induced sepsis.^25^ Also observed in intracellular parasites as the inhibition of lysosomal fusion, a classical escape mechanism was observed after infection by *Mycobacterium*, *Chlamydia*, *Toxoplasma* and *Trypanosoma cruzi*, for example. Another way that pathogens can prolong their survival inside the host is by prevention of host-cell apoptosis and by the modulation of reactive oxygen and nitrogen species generation.^5^ Szczepanski et al.^26^ revealed that canine respiratory coronavirus enters HRT-18G cells via the caveolin-1 dependent pathway. Thus, these previous results support the notion that caveolin-1 might play a role in the interaction of *S. agalactiae* and HUVEC cells.

Serine/threonine kinase Akt is activated by G protein-coupled receptors that induce the production of phosphatidylinositol (3,4,5) trisphosphate (PIP3) by PI3K.^10^ In this work, we demonstrated that the integrity of lipid rafts microdomains and the activity of PI3K/Akt are required for invasion of GBS90356 strain to human endothelial cells. Indeed, the phosphorylation of Akt and PI3K were suppressed by cholesterol depletion using MβCD, suggesting that membrane microdomain integrity is important for PI3K/Akt activation during *S. agalactiae* infection. Peres et al.^27^ showed that membrane microdomains were an essential site for PI3K activation during lysophosphatidic acid stimulation in Vero cells. Lipid rafts also induced platelet aggregation via PI3K-dependent Akt phosphorylation by stromal cell-derived factor-1α signaling.^10^

We cannot exclude the possibility that the results obtained in our studies could be specific to *S. agalactiae* type III belonging to the hypervirulent ST-17, and that other capsular type III strains could behave in different ways. More studies are necessary to unravel this possibility.

Our results demonstrate that lipid microdomain affects *S. agalactiae* recognition by HUVEC through PI3K/Akt signaling pathway. In addition, *S. agalactiae* cytoadhesion using membrane microdomains suggests a selective role of lipid raft molecules, such as flotillin-1 and caveolin-1. Hence, an understanding of the role of host membrane rafts in *S. agalactiae* invasion may shed light on the molecular mechanisms of infection.
